# Association of COMT genotypes with S-COMT promoter methylation in growth-discordant monozygotic twins and healthy adults

**DOI:** 10.1186/1471-2350-12-115

**Published:** 2011-09-01

**Authors:** Felix Schreiner, Osman El-Maarri, Bettina Gohlke, Sonja Stutte, Nicole Nuesgen, Manuel Mattheisen, Rolf Fimmers, Peter Bartmann, Johannes Oldenburg, Joachim Woelfle

**Affiliations:** 1 Pediatric Endocrinology Division, Children's Hospital, University of Bonn, Adenauerallee 119, Bonn 53113, Germany; 2 Institute for Experimental Hematology and Transfusion Medicine, University of Bonn, Sigmund-Freud-Str. 25, Bonn, 53127, Germany; 3 Institute for Medical Biometry, Informatics and Epidemiology, University of Bonn, Sigmund-Freud-Str. 25, Bonn, 53127, Germany; 4 Department of Neonatology, Children's Hospital, University of Bonn, Adenauerallee 119, Bonn 53113, Germany

## Abstract

**Background:**

Catechol-O-Methyltransferase (COMT) plays a key role in dopamine and estrogen metabolism. Recently, COMT haplotypes rather than the single polymorphism Val158Met have been reported to underlie differences in protein expression by modulating mRNA secondary structure. So far, studies investigating the epigenetic variability of the S-COMT (soluble COMT) promoter region mainly focused on phenotypical aspects, and results have been controversial.

**Methods:**

We assessed S-COMT promoter methylation in saliva and blood derived DNA with regard to early pre- and postnatal growth as well as to genotype for polymorphisms rs6269, rs4633, and rs4680 (Val158Met) in 20 monozygotic twin pairs (mean age 4 years), who were discordant for intrauterine development due to severe feto-fetal-transfusion syndrome. Methylation levels of two previously reported partially methylated cytosines were determined by the quantitative SIRPH (SNuPE- IP RP HPLC) assay.

**Results:**

Overall, we observed a high variability of S-COMT promoter methylation, which did not correlate with individual differences in the pre- or postnatal growth pattern. Within the twin pairs however we noted a distinct similarity that could be linked to underlying COMT genotypes. This association was subsequently confirmed in a cohort of 93 unrelated adult controls. Interestingly, 158Val-alleles were found at both ends of the epigenotypical range, which is in accordance with a recently proposed model of COMT haplotypes corresponding to a continuum of phenotypical variability.

**Conclusion:**

The strong heritable component of S-COMT promoter methylation found in our study needs to be considered in future approaches that focus on interactions between COMT epigenotype and phenotype.

## Background

Catechol-O-Methyltransferase (COMT) plays a key role in dopamine and estrogen metabolism. In humans, the COMT gene on chromosome 22q11.21 encodes two different protein isoforms, MB-COMT (membrane bound) and S-COMT (soluble isoform), each with its own promoter [1]. The influence of a functional SNP 158Met/Val (rs4680), which is located within the coding region of both MB-COMT and S-COMT, has been investigated in a broad spectrum of psychiatric diseases and estrogen-dependent disorders [2-4]. However, reported genotype effects were often modest or even inconsistent, suggesting the presence of additional functional SNPs within this gene. Indeed, Diatchenko and co-workers identified three common COMT haplotypes corresponding to a continuum of phenotypical variability, namely pain sensitivity, with the 158Val allele being part of both the high- and low-sensitive haplotype, whereas the haplotype containing 158Met associates with average pain sensitivity [5]. More recently, the same group demonstrated that these three haplotypes mediate their functional significance by alteration of mRNA secondary structure such that the most stable structure associates with the lowest protein level and activity [6].

Besides heritable genetic factors, environmental events which accumulate over lifetime are a second major determinant of an individual's gene expression pattern which contributes to both physiological appearance and disease disposition. The mechanisms behind this intra-generational adaptation are complex and involve epigenetic processes such as chromatin modification and DNA methylation [7,8]. In mammals, DNA methylation occurs almost exclusively at the cytosine of CG dinucleotides, and several methods to quantify methylation at these elements have been established in recent years [9,8-11]. Since then, first studies analyzing COMT promoter methylation in relation to phenotypical aspects have been published. Sasaki et al. reported on MB-COMT promoter methylation in endometrial cancer but not in normal endometrial tissue; in contrast, the S-COMT promoter was found to be unmethylated in both cancerous and normal endometrium [12]. Partial methylation of the MB-COMT promoter was also reported in human frontal lobe brain tissues, and hypomethylation at this area was observed in schizophrenia and bipolar disorder patients [13]. Although no such clear association between methylation levels and disposition to schizophrenia was found, Murphy et al. detected two CG sites in the S-COMT promoter region with only partial methylation in both blood and brain tissues [14]. Subsequently, Mill et al. analyzed methylation levels at these two CG sites in a cohort of monozygotic twins with discordant birth weight. Interestingly, the authors report on a considerable variation in the concordance of methylation levels between the twin pairs, which however did not associate with auxological variables at birth [15].

Since discordant birth weight in monozygotic twins can arise from several pathologies, we decided to assess S-COMT promoter methylation levels in a cohort of discordant monozygotic twins that have been selected for severe feto-fetal-transfusion syndrome (FFTS). This condition is caused by anastomosing placental vessels that result in unequal blood supply and thus substantial differences in fetal nutrition between donor and recipient. In animal models, the strategy of nutrient restriction is increasingly used to investigate long-term epigenetic effects of intrauterine growth retardation [16-18]. In our cohort, we observed a remarkable variation in S-COMT promoter methylation levels in both blood and saliva derived DNA, which did not correlate with pre- or postnatal growth parameters. On the other hand, our data provide evidence for a strong association of S-COMT promoter methylation with the underlying COMT genotype.

## Methods

### Subjects

For an ongoing study that focuses on epigenetic parameters with respect to adverse intrauterine environment we selected 20 Caucasian twin pairs with a discordant intrauterine growth pattern due to severe feto-fetal transfusion syndrome (FFTS). In all 20 pregnancies, this condition had been diagnosed by mid-trimester ultrasound examinations, and endoscopic laser coagulation of the anastomosing placental vessels was successfully performed before 25 weeks of gestation in a single center (Prof. Dr. K. Hecher, Barmbek Hospital, Hamburg, Germany). Details on survival rates and early neonatal outcome of this treatment regime are described elsewhere [19]. All children were born in the local referring hospital.

Mean age at birth was 34.8 weeks of gestation (SD ± 2.1 wks; range 29.7-37.4 wks). Mean birth weight of the entire cohort was 1970 g (SD ± 500 g; range 790-3060 g). Birth weight differences between donor and recipient ranged from 0 to 62% (mean 20.5%). Since FFTS occurs exlusively in monozygotic twin pregnancies, no further genetic examinations were necessary to confirm their monozygosity. 38 of 40 children of the present cohort did not suffer from severe postnatal complications. Two children show a significant delay of psychomotor development after major intracranial bleeding shortly after birth; auxological and epigenetic measurements of these two children were thus interpreted with caution. On examination, mean age of the cohort was 4.4 years (SD ± 0.6 yrs; range 2.7-5.1 yrs).

Auxological parameters including calculations of intra-twin pair differences were expressed as SD-scores according to national reference percentiles for singleton children [20,21]. At any time, parameters between former donor and recipient were classified as discordant if difference in SD scores was 1.0 or higher (except for birth weight, which is not normally distributed; birth weight discordance was defined if difference was > 10%).

Unrelated control individuals (93 adult blood donors; 47 females) were recruited from the blood donation unit of the Institute for Experimental Hematology and Transfusion Medicine, University of Bonn. Written informed consent was obtained from all participants or their parents. The study was approved by the ethic's committee of the University of Bonn.

### DNA samples and bisulfite conversion

DNA from blood lymphocytes was extracted using the QiaAmp^® ^DNA purification kit (Qiagen, Germany). Buccal cell derived DNA was collected and prepared from Oragene^® ^saliva samples according to the manufacturer protocol (DNA Genotek, Canada). Suitable amounts of saliva were obtained from 17 out of 20 twin pairs, while blood-derived DNA was available from all 40 children. For methylation analysis, a total of 1 μg DNA was modified by bisulfite conversion using the Epitect^® ^kit (Qiagen, Germany). The basic principle of bisulfite modification is the chemical conversion of unmethylated cytosine residues to uracil, whereas methylated cytosines remain unchanged [22]. This step allows accurate quantitative measurement of locus-specific cytosine methylation by several PCR-based downstream reactions [9,10,23].

### Quantitative methylation analysis

**Figure 1:**
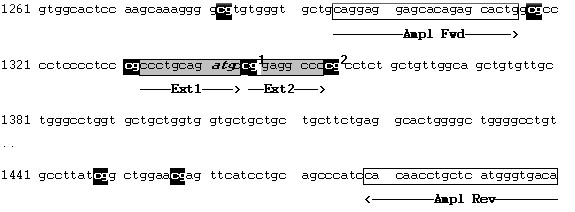
**Genomic sequence flanking the S-COMT promoter region (atg in italics)**. Primer sequences for the PCR amplification of bisulfite-converted DNA are Ampl Fwd: 5' TAGGAGGAGTATAGAGTATTG 3' and Ampl Rev: 5' TATCACCCATAAACAAATTATA 3'; Ext1 and Ext2 bordering CG1 and CG2 indicate the complementary extension primer sequences used in the SNuPE analysis.

**Figure 2:**
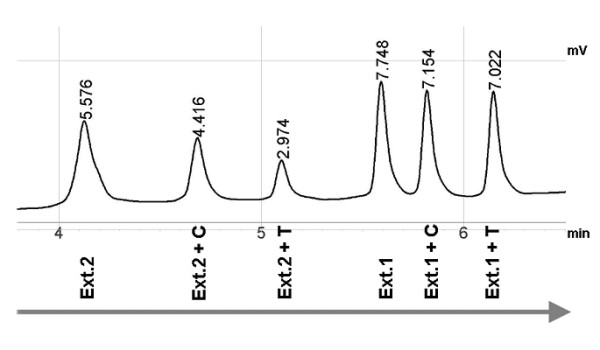
**A SNuPE IP RP HPLC example showing the discrimination of extension primer only vs. extended primer oligonucleotides (T with higher molecular weight than C)**. Methylation levels can be calculated by comparison of the relative heights of Ext.+C (formerly methylated cytosine) and Ext.+T (formerly unmethylated cytosine). Differences in length and composition of the two extension primers allowed discrimination of two separate extension primer sets (Ext.1 and Ext.2) within one SNuPE run.

Site-specific methylation was determined at two CG dinucleotides within the S-COMT promoter region (Figure 1) which have been found to be partially methylated in two previous studies [14,15]. A detailed description of the SIRPH (SNuPE IP RPHPLC) assay used is given elsewhere [23]. Briefly, a single nucleotide primer extension reaction (SNuPE) of bisulfite converted DNA in combination with ion pair reverse phase high performance liquid chromatography (IP RP HPLC) enables discrimination and quantification of methylated and unmethylated CGs based on the respective mass and hydrophobicity of the extended primer product. An example demonstrating the chromatographic discrimination between methylated and unmethylated cytosines is given in Figure 2. All measurements were done in duplicate, including repeated DNA extraction and bisulfite conversion. Variation between the two runs was thoroughly below 5%. Methylation levels given in the results section represent the respective average values.

A potential amplification bias due to additional SNPs that would cause mismatch of amplification and/or extension primers was ruled out by genomic sequencing of the surrounding regions in all 20 twin pairs. We did not detect any further sequence variation in our DNA samples. A screen of online databases revealed only one SNP (rs72563160) within the primer complementary regions. This variant is found with a low frequency (0.04) in African populations and seems to be absent in Caucasians http://www.ncbi.nlm.nih.gov/projects/SNP. Oligonucleotide sequences for PCR and SNuPE reactions are available on request.

### Genotyping

Genotypes for the rs4680 variant (Val158Met) were analyzed by PCR-RFLP, using an NlaIII restriction digest as described previously [24]. Genotypes for rs6269 (-91 bp to ATG) and rs4633 (in twins only; +186 bp to ATG) were determined by direct genomic sequencing. This method was chosen primarily in order to exclude additional sequence variation within or bordering to the amplified region.

Based on the genotype information for these three SNPs, we constructed haplotypes to each individual. According to the findings of Diatchenko and coworkers [5,6], about 95% of the alleles in the Caucasian population carry three major functional haplotypes which are formed by four SNPs. We are aware that we have analyzed only three (rs6269-A/G, rs4633-C/T, rs4680-A/G) of these four variants (lacking rs4818-C/G) in twins, and only two (rs6269-A/G, rs4680-A/G) in blood donors. However, determination of the latter two genotypes is sufficient to differentiate between the three common COMT activity haplotypes [25]. Assessment of individual haplotypes in twins and blood donors was performed by the FAMHAP software [26]. Haplotype frequencies in both cohorts (*twins: *LPS 0.450, APS 0.525, HPS 0.025; *blood donors: *LPS 0.385, APS 0.516, HPS 0.093) were comparable to those reported previously in Caucasian individuals [6].

### Statistical analysis

Data analysis was performed using the SPSS software version 17.0. To assess the degree of association between methylation levels and/or auxological parameters within twin pairs and between groups, we used Pearson correlation coefficients. Inter-twin-pair differences were analyzed using Student's t-tests, intra-twin-pair differences using a paired t-test. Backward stepwise multivariate linear regression analysis was performed to identify the independent variables that best predicted methylation levels.

Testing for differences in the degree of methylation between genotypes was performed using one-way ANOVAs and t-tests. Since both methods assume that all observations are independent, which for methylation values of MZ twins might not be the case, we re-calculated the differences between the genotype groups regarding COMT methylation in blood and saliva using a mixed linear model with methylation and genotype as two fixed factors and with family as a random factor to account for within-family dependence. In addition, under the assumption of an additive mode of inheritance we tested an additive genetic model (1df test) using linear regression analysis by coding genotype information as 0, 1, or 2 copies of the minor allele for each SNP, respectively.

A p-value < 0.05 was considered statistically significant; a p-value between 0.05 and 0.1 as an indicator for a statistical trend of significance.

## Results

**Figure 3:**
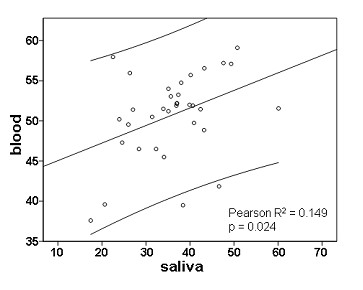
**Intra-individual correlation between saliva and blood DNA methylation levels at the S-COMT promoter (mean methylation = (CG1 + CG2)/2))**.

Among the twin samples, we observed a high variability of individual methylation levels at both analyzed CG sites. In blood derived DNA, levels ranged from 26.5 to 48.3% (mean 40.1 ± 5.4%) at CG1 (CG 23 in reference 14) and from 48.7 to 70.1% (mean 61.9 ± 6.1%) at CG2 (CG 27 in reference 14). In saliva DNA, levels ranged from 13.9 to 51.3% (mean 30.2 ± 8.2%) at CG1 and from 21.0 to 68.0% (mean 41.8 ± 10.6%) at CG2. In both specimens there was a strong correlation between methylation levels at these two CG dinucleotides (CG1 *vs*. CG2: R = 0.94, p < 0.001 for saliva DNA; R = 0.95, p < 0.001 for blood DNA). This is in accordance with the findings of Mill et al. [15], who also reported on a relatively high correlation coefficient (R = 0.77) of methylation levels at these two CG sites in saliva derived DNA. In addition, we found a significant intra-individual correlation between blood *vs*. saliva DNA methylation levels (R = 0.386, p = 0.024; Figure 3).

**Figure 4:**
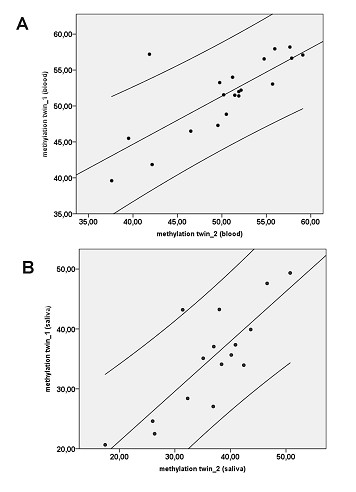
**Intra-twinpair correlation between both twins for blood- (A) and saliva-derived DNA (B)**. Note that in (B) one pair of outliers was not included in the analysis.

Finally, we observed a remarkable intra-twin pair correlation of mean S-COMT methylation levels (donor *vs*. recipient: R = 0.77, p < 0.001 in blood DNA and R = 0.41, p = 0.106 in saliva DNA; the latter coefficient increased to R = 0.80, p < 0.001 after exclusion of one pair with an exceptionally high intra-pair variation; see Figure 4). These findings led us to assume a strong heritability of individual S-COMT promoter methylation levels.

### S-COMT promoter methylation and genetic background

We determined genotypes for the variants rs6269-A/G, rs4633-C/T, and rs4680-A/G for all twin pairs and compared S-COMT promoter methylation levels among the genotype groups. In blood derived DNA, we found highly significant associations of methylation levels with each of the analyzed genotypes (table 1). Testing for differences was performed using one-way ANOVAs and t-tests. Since both methods assume that all observations are independent, which for methylation values of MZ twins might not be the case, we re-calculated the differences between the genotype groups regarding COMT methylation in blood and saliva using a mixed linear model. This did not change the observed significance levels for methylation differences between the respective genotypes in blood samples. In saliva DNA, variation of methylation for both analysed CGs was markedly higher, and mean methylation levels between the genotype groups differed with only weak significance (table 2) when distinguishing between two genotype groups; the analysis between all three groups using the mixed linear model did not reveal significant differences. In addition, under the assumption of an additive mode of inheritance we tested an additive genetic model, which confirmed the significance levels between genotype groups for blood DNA in both cohorts (table 1/3) and revealed trends for the saliva samples (rs6269: p = 0.086; rs4633: p = 0.070; rs4680: p = 0.070).

**Table 1:** S-COMT methylation [% ± SD] with respect to COMT genotype in Blood DNA (twin cohort)

Variant	CG1	CG2b	mean	CG1	CG2b	mean	CG1	CG2b	mean	p-value
**rs6269**	**AA **44.8 ± 3.1	67.1 ± 3.1	**56.0 ± 3.0 **(n = 10)	**AG **40.3 ± 3.0	62.1 ± 3.9	**51.2 ± 3.4 **(n = 24)	**GG **30.6 ± 3.0	51.4 ± 3.0	**41.0 ± 2.7 **(n = 6)	< 0.01

**rs4633**	**CC **33.2 ± 5.3	54.2 ± 5.8	**43.7 ± 5.4 **(n = 8)	**CT **40.3 ± 3.2	62.0 ± 4.1	**51.2 ± 3.5 **(n = 22)	**TT **44.8 ± 3.1	67.1 ± 3.1	**56.0 ± 3.0 **(n = 10)	< 0.01

**rs4680**	**AA **44.8 ± 3.1	67.1 ± 3.1	**56.0 ± 3.0 **(n = 10)	**AG **40.3 ± 3.2	62.0 ± 4.1	**51.2 ± 3.5 **(n = 22)	**GG **33.2 ± 5.3	54.2 ± 5.8	**43.7 ± 5.4 **(n = 8)	< 0.01

Note that similar or equal methylation values across genotype groups for different SNPs result from a high (in the twin cohort almost complete) linkage disequilibrium between rs6269, rs4633, and rs4680. Please note that shown p-values were calculated using one-way ANOVAs, which do not take the interrelatedness of the observations into account.

**Table 2:** S-COMT methylation [% ± SD] with respect to COMT genotypes in Saliva DNA (twin cohort)

Variant	CG1	CG2b	mean	CG1	CG2b	mean	CG1	CG2b	mean	p-value
**rs6269**	**AA **34.7 ± 14.7	43.4 ± 18.0	**39.0 ± 16.2 **(n = 10)	**AG **30.9 ± 6.1	40.5 ± 7.8	**35.7 ± 6.8 **(n = 24)	**GG **23.1 ± 9.3	31.9 ± 11.9	**27.5 ± 10.6 **(n = 6)	< 0.05 *

**rs4633**	**CC **24.7 ± 8.2	33.2 ± 10.1	**28.9 ± 9.1 **(n = 8)	**CT **31.2 ± 6.2	41.0 ± 7.8	**36.1 ± 6.8 **(n = 22)	**TT **34.7 ± 14.7	43.4 ± 18.0	**39.0 ± 16.2 **(n = 10)	< 0.05 *

**rs4680**	**AA **34.7 ± 14.7	43.4 ± 18.0	**39.0 ± 16.2 **(n = 10)	**AG **31.2 ± 6.2	41.0 ± 7.8	**36.1 ± 6.8 **(n = 22)	**GG **24.7 ± 8.2	33.2 ± 10.1	**28.9 ± 9.1 **(n = 8)	< 0.05 *

* rs6269: AG *vs*. GG p < 0.05; rs4633: CC *vs*. CT p < 0.05; rs4680: AG *vs*. GG p < 0.05. Please note that shown p-values were calculated using t-tests, which does not take the interrelatedness of the observations into account.

**Table 3:** S-COMT methylation [% ± SD] with respect to COMT genotype in Blood DNA (blood donors)

Variant	CG1	CG2b	mean	CG1	CG2b	mean	CG1	CG2b	mean	p-value
**rs6269**	**AA **47.0 ± 4.6	60.1 ± 3.7	**53.6 ± 3.5 **(n = 26)	**AG **42.2 ± 4.7	55.7 ± 5.1	**49.0 ± 4.8 **(n = 44)	**GG **36.6 ± 4.6	51.4 ± 5.3	**44.0 ± 4.8 **(n = 23)	< 0.01

**rs4680**	**AA **47.3 ± 4.2	60.3 ± 3.7	**53.8 ± 3.4 **(n = 33)	**AG **40.8 ± 3.8	54.7 ± 4.5	**47.8 ± 4.0 **(n = 46)	**GG **34.1 ± 4.1	49.1 ± 5.4	**41.6 ± 4.5 **(n = 14)	< 0.01

Note that due to the high linkage disequilibrium between rs6269, rs4633, and rs4680, in the replication cohort we assessed only rs6269 and rs4680, which are sufficient to differentiate between the 3 common activity haplotypes.

Because any additional sequence variation would have to be considered as a potential source of amplification bias in the SNuPE analysis, we excluded the presence of additional SNPs by direct genomic sequencing in all 20 twin pairs. We did not detect any further sequence variation.

Next, we analyzed S-COMT promoter methylation and COMT genotypes in an additional sample of 93 healthy adult blood donors. Methylation levels at both cytosines (lymphocyte DNA only; CG1 mean 42,2 ± 5,9%; CG2 mean 55,9 ± 5,7%), range of variation, as well as genotype-epigenotype associations (all p < 0.01) were comparable to those seen in the twin cohort. We did not detect any significant changes in the methylation pattern that may be related to the considerable age difference between the two cohorts.

**Figure 5:**
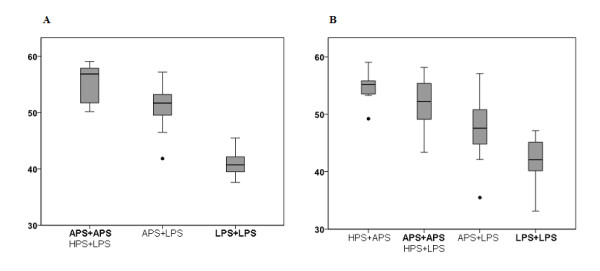
**Mean S-COMT methylation levels with respect to the presumed COMT haplotype carrier status (A = twin cohort, B = adult controls)**. In both cohorts, no individuals homozygous for the HPS haplotype were found. All other haplotype combinations were ranked within an imaginary scale corresponding to the proposed phenotypical differences (high > average > low pain sensitive, according to [Nackley et al., 2006]).

Considering the functional relevance of three major COMT haplotypes due to variation in the mRNA secondary structure and differences in protein expression, we were interested to see whether promoter methylation levels parallel the proposed phenotypical grading into low- (LPS), average- (APS), and high-pain-sensitive (HPS), respectively [5,6]. Figure 5 shows mean S-COMT promoter methylation levels with regard to the assumed haplotypes and displays the putative association in a graphical way. Belonging to both the high- and the low-pain-sensitive haplotype, rs4680 mutant alleles (158Val) were found at both ends of the epigenotypical range.

### Pre-and postnatal growth, COMT-genotypes and S-COMT promoter methylation

In order to determine a potential impact of environmental factors on the individual S-COMT promoter methylation, we compared methylation levels with pre- and postnatal growth parameters. Concerning the FFTS-status, we did not detect differences in methylation levels between donors and recipients (see table 4), even when analyzing only the subgroup of twin pairs with discordant birth weight (10 pairs with a difference of > 10%, data not shown).

**Table 4:** Auxiological and epigenetic differences between donors and recipients (mean ± SEM)

	Donors	Recipients	p-value
**Birth weight SDS**	-1.45 ± 0.2	-0.64 ± 0.2	< 0.01

**Birth length SDS**	-1.6 ± 0.3	-0.5 ± 0.2	< 0.01

**Height SDS (follow-up)**	-0.8 ± 0.2	-0.3 ± 0.2	< 0.01

**BMI SDS (follow-up)**	-0.7 ± 0.2	-0.1 ± 0.2	< 0.01

**S-COMT methylation (blood)**	50.4 ± 1.4	51.6 ± 1.2	n.s.

**S-COMT methylation (saliva)**	36.5 ± 2.1	35.0 ± 2.2	n.s.

We next aimed to directly correlate auxological parameters with methylation levels. Disregarding the information on the strong heritability of this locus' methylation pattern, we observed a significant correlation between S-COMT promoter methylation levels and the timing of laser treatment (r = 0.448; p = 0.006), but no significant correlations regarding methylation levels *vs*. gestational age or auxological birth parameters (birth weight, length, head circumference). However, COMT genotypes were not equally distributed among gestational ages at the time of laser treatment (table 5) as well as at birth (table 6). After correction for background genetics using linear regression analysis, only genotype (β = -9.5; p < 0.001) but not timing of laser treatment or gestational age at birth contributed significantly to the observed variance of COMT methylation.

**Table 5:** Genotype and gestational age at laser treatment (mean ± SD)

	Wks	wks	wks	p-value
**rs6269**	**AA **22.4 ± 1.2 (n = 10)	**AG **20.5 ± 2.4 (n = 24)	**GG **18.8 ± 0.5 (n = 6)	< 0.01

**rs4633**	**CC **18.8 ± 0.4 (n = 8)	**CT **20.7 ± 2.5 (n = 22)	**TT **22.4 ± 1.2 (n = 10)	< 0.01

**rs4680**	**AA **22.4 ± 1.2 (n = 10)	**AG **20.7 ± 2.5 (n = 22)	**GG **18.8 ± 0.4 (n = 8)	< 0.01

**Table 6:** COMT genotype and gestational age at birth (mean ± SD)

	Wks	wks	Wks	p-value
**rs6269**	**AA **36.2 ± 0.9 (n = 10)	**AG **34.2 ± 2.2 (n = 24)	**GG **35.0 ± 1.9 (n = 6)	0.082 *

**rs4633**	**CC **33.7 ± 2.9 (n = 8)	**CT **34.6 ± 1.8 (n = 22)	**TT **36.2 ± 0.9 (n = 10)	0.089 *

**rs4680**	**AA **36.2 ± 0.9 (n = 10)	**AG **34.6 ± 1.8 (n = 22)	**GG **33.7 ± 2.9 (n = 8)	0.089 *

* Kruskal-Wallis test significance levels are displayed in the table;

Mann-Whitney-U-test: rs6269: AA *vs *AG p = 0.034; rs4633: CC *vs*. TT p = 0.049; rs4680: AA *vs*. GG p = 0.049.

At birth intra-twin-pair differences were greater than 1.0 SDS in 10/19 for birth length (data missing in one pair) and greater than 10% in 10/20 pairs for birth weight, respectively. After 4 years only 4 twin pairs were still discordant in length-SDS. We compared both the delta-SD scores (height) in intra-twin-pair approximation and individual height within the total sample of 40 children with measurements of absolute and delta S-COMT methylation. Both with and without correction for genotypes, we did not see any significant association between postnatal growth parameters and methylation levels (p > 0.05 in all analyses). As mentioned above, two children suffered from intracranial bleeding und a subsequent delay in motor-development. Postnatal growth of these two children was obviously impaired, whereas S-COMT promoter methylation appeared to be unaffected by this condition as it was well within the intra-twin-pair range seen in the remaining pairs.

Finally, we detected a tendency towards slightly higher methylation levels in males (mean S-COMT promoter methylation 48.5 +/- 5.8% in females versus 53.0 +/- 4.8% in males; p = 0.01). Similar differences have been reported previously for other non-imprinted loci [27]. Inclusion of gender in linear regression analysis did not affect genotype-epigenotype associations in twins. In blood donors, inclusion of gender in the linear regression analysis led to an increase of R^2 ^from 0.534 to 0.609, explaining an additional 7.5% of variance. Therefore we assume that the overall confounding effect of gender is small compared to the association with COMT genotypes.

## Discussion

Based on two studies reporting on partial methylation of two CG sites within the S-COMT promoter region [14,15], we aimed to determine whether and to which extent environmental and genetic variables associate with the individual epigenetic profile at the S-COMT promoter in a cohort of discordant monozygotic twins. Our findings provide evidence for a strong interaction between genetic background and S-COMT promoter methylation. Considering the well recognized functional relevance of discrete genomic variation and the physiological role of locus-specific cytosine methylation in the context of transcription and gene expression, such an association is not surprising. Indeed, there are several recent reports on locus-specific methylation patterns that are closely associated with the genetic background [28-33]. Moreover, we did not detect any significant association between methylation levels and pre- or postnatal growth parameters and could confirm the close relation between genotype and epigenotype in a separate cohort of adult singleton blood donors. Interestingly, methylation levels in 4-year-old children and adults were largely comparable, which further underlines the close relation between genetic background and promoter methylation at this locus.

While planning this study we were aware that methylation analyses using peripheral blood derived DNA in monozygotic twins may be biased due to their fused circulations. Fetal sharing of circulating blood has been described in monozygotic twin pregnancies even without the presence of FFTS [34]. In a study on monocygotic twins discordant for Beckwith-Wiedemann syndrome, Weksberg et al. reported on imprinting abnormalities in hematopoietic cells from both the affected and the unaffected twin, whereas in fibroblasts epigenetic defects were found in the affected twin only [35]. Thus we decided to analyze DNA from two tissues: blood and oral epithelial cell samples. Preceding laboratory tests using conventional buccal smears revealed a high degree of variation regarding both amount and purity of DNA, which may result from bacterial contamination and/or DNA degradation even during short term storage of these specimens. Accordingly, methylation measurements in smears taken from the same individual at the same time were scarcely reproducible (data not shown). In contrast, methylation measurements using DNA from Oragene liquid saliva samples revealed a markedly higher intra-individual reproducibility. This may explain why Mill et al. who exclusively analyzed S-COMT methylation in buccal smear DNA failed to detect a similar heritability in their twin cohort [15]. On the other hand, this group had selected their twins primarily on the presence of a significant discordance in birth weight. Although they do not give detailed information on the underlying pathology, we speculate that the majority of these twin pairs suffered from conditions that worsened in the last trimester of pregnancy, in contrast to an equalization of the formerly disproportionate (mid-trimester) blood supply in our FFTS twins. Thus, differences in the plasticity of the (S-COMT promoter) epigenetic encoding during different periods of the intrauterine development might explain this discrepancy, too.

We were not able to detect significant differences in S-COMT promoter methylation levels between donors and recipients. This seems to indicate that the adverse intrauterine environment due to FFTS until decompensation and laser treatment did not influence formation and/or maintenance of the promoter methylation at this region. Within the period after laser treatment however auxological parameters converged in the majority of twin pairs, meaning that critical differences in placental supply had been dissolved. Consequently, a potential impact of malnutrition during later gestational phases may be missed by our approach.

In order to eliminate any amplification bias that may arise from the occurrence of single nucleotide polymorphisms within or nearby the primer regions, we sequenced the S-COMT promoter region in genomic DNA samples from all 20 twin pairs and did not detect any new sequence variation. Another potential source of biased methylation analysis however remains the lack of information on the leukocyte subtype distribution in the blood samples used for DNA extraction. This issue has already been discussed by Boks et al. in their recent publication on methylation levels of several CG loci found to be closely linked to genetic polymorphisms in a large-scale twin study using the Illumina GoldenGate assay [33]. Although we do not have current leukocyte subtype counts, the conserved (albeit less clear) genotype-epigenotype-association found in the Oragene saliva samples seems to rule out this objection.

A limitation of our study is that we do not have quantitative mRNA measurements, which would allow us to evaluate our findings with respect to the physiological entity of promoter methylation and transcriptional activity. In vitro experiments by Nackley and co-workers using transfected full-length COMT cDNA that differed only in three nucleotides within the coding region of exons 1 and 2 revealed marked differences in the levels of protein expression and enzyme activity which could be explained by either differences in mRNA secondary structure stability or, to less extent, a previously reported decrease in protein thermostability mediated by the 158Met allele. Total COMT RNA abundance, in contrast, did not parallel COMT protein levels, most likely attributing to the nature of their experimental design in that COMT was expressed in vitro under the regulation of identical promoters [6]. Our data provide evidence that a set of polymorphisms within the promoter and coding region of this gene strongly associates with the methylation status of the (S-COMT-) promoter region. Interestingly, individuals assumed to carry two alleles with the LPS haplotype, which was previously associated with the relatively highest S-COMT enzyme activity (figure one in ref. [6]), exhibited a markedly lower S-COMT promoter methylation as compared to those carrying the APS and HPS alleles (Figure 5). Presuming an inverse relation between promoter methylation and transcriptional activity, a constellation of lower promoter methylation in alleles associated previously with higher protein activity and *vice versa *would conflict with the assumption of a simple feed-back response between transcriptional activity and the abundance of either COMT protein or its substrates. Indeed, besides an estrogenic down-regulation of COMT expression mediated by estrogen response elements that have been found in the promoter regions of both the proximal S-COMT and the distal MB-COMT promoter [36,37], there is also evidence for an induction of COMT by estradiol and ethinyl-estradiol which would be consistent with the assumption of a physiological response to a threat to the genome by catechol-estrogens that can be blocked by their O-methylation [36,38].

To our knowledge, the only comprehensive investigation so far that aimed to analyze COMT epigenotype, genotype, and phenotype including mRNA expression data is the study of Abdolmaleky et al. [13]. The authors report on MB-COMT promoter hypomethylation with a concomitant increase in MB-COMT transcription in frontal lobe tissue from schizophrenia and bipolar disorder patients, as compared to unaffected controls. Except for a borderline significance found for the enrichment of 158Val alleles with MB-COMT promoter hypomethylation and disease disposition, there was however no consistent relation between individual epigenotype and the Val158Met polymorphism. It will be interesting to test whether the inclusion of further genetic variants may strengthen the relation between genotype and epigenotype as seen for the S-COMT promoter methylation in lymphocytes in our sample.

## Conclusion

Our data revealed a strong heritable component of individual S-COMT promoter methylation. Simultaneous consideration of heritable, i.e. genetic components should be a basic item in all studies that focus on the relation between phenotype and epigenotype.

## Disclosure statement

The authors declare that they have no competing interests.

## Authors' contributions

FS, OEM, BG, PB, JO, and JW designed the study. FS, BG, and SS collected patient data and samples. FS and NN performed the experiments. FS, OEM, MM, RF, and JW analyzed the data. FS and JW wrote the paper. All authors read and approved the final manuscript.

## Pre-publication history

The pre-publication history for this paper can be accessed here:

http://www.biomedcentral.com/1471-2350/12/115/prepub
